# High biogeographic and latitudinal variability in gastropod drilling predation on molluscs along the eastern Indian coast: Implications on the history of fossil record of drillholes

**DOI:** 10.1371/journal.pone.0256685

**Published:** 2021-08-26

**Authors:** Subhronil Mondal, Hindolita Chakraborty, Sandip Saha, Sahana Dey, Deepjay Sarkar

**Affiliations:** 1 Department of Earth Sciences, Indian Institute of Science Education and Research (IISER) Kolkata, Mohanpur, West Bengal, India; 2 Department of Geology, University of Calcutta, Kolkata, India; 3 Geological Studies Unit, Indian Statistical Institute, Kolkata, India; 4 Department of Geology, Triveni Devi Bhalotia College Raniganj, West Bengal, India; Naturhistoriska Riksmuseet, SWEDEN

## Abstract

Studies on the large-scale latitudinal patterns of gastropod drilling predation reveal that predation pressure may decrease or increase with increasing latitude, or even show no trend, questioning the generality of any large-scale latitudinal or biogeographic pattern. Here, we analyze the nature of spatio-environmental and latitudinal variation in gastropod drilling along the Indian eastern coast by using 76 samples collected from 39 locations, covering **~**2500 km, incorporating several ecoregions, and ~15° latitudinal extents. We find no environmental or latitudinal gradient. In fact, drilling intensity varies highly within the same latitudinal bin, or oceanic sub-basins, or even the same ecoregions. Moreover, different ecoregions with their distinctive biotic and abiotic environmental variables show similar predation intensities. However, one pattern is prevalent: some small infaunal prey taxa, living in the sandy-muddy substrate—which are preferred by the naticid gastropods—are always attacked more frequently over others, indicating taxon and size selectivity by the predators. The result suggests that the biotic and abiotic factors, known to influence drilling predation, determine only the local predation pattern. In the present case, the nature of substrate and prey composition determines the local predation intensity: soft substrate habitats host dominantly small, infaunal prey. Since the degree of spatial variability in drilling intensity within any time bin can be extremely high, sometimes greater than the variability across consecutive time bins, temporal patterns in drilling predation can never be interpreted without having detailed knowledge of the nature of this spatial variability within a time bin.

## Introduction

Studies on spatio-temporal and latitudinal patterns of gastropod drilling predation on the molluscan prey have been a prevalent theme of research interest in the last few decades [1 and references therein]. Traces produced by this biological interaction, which can be preserved as circular or semi-circular holes on the prey shell, has a very rich fossil record ranging from the Mesozoic onwards [2–4]. By quantifying the intensity of this trace–which gives us a measure of predation intensities–many macroecological and macroevolutionary hypotheses have been tested [3,5–10]. Based on these studies, it is known that the gastropod drilling predation was low during most of the Mesozoic, after which it intensified globally [2,3]. While this rise in predation pressure through time may be the result of increasing competitive interaction among the predators and their prey [see 11 for review], some short-term, local ‘noises’ could potentially detrend the global, long-term pattern [see 12]. Two such common noises are uneven sampling and local environmental variations in drilling [8,13,14].

In terms of sampling, although the history of drilling predation is known based on numerous publications, an actual analysis of the quality of drilling predation fossil record reveals its scarce, incomplete nature–data are temporally and latitudinally highly skewed [10,15]. The same pattern can be seen for the Recent gastropod drilling records—compared to the reports of the fossil record of drilling, reports of predation from the Recent oceans are very scarce. The nature of this scarce record can be categorized into the following: (1) most data come from the USA and Europe, and rarely from South America [see 8] and Japan [16–21], missing other biogeographic regions globally; (2) most data are restricted to the Atlantics, and eastern Pacific, whereas data from the Indo-Western Pacific are rare; (3) even hemispherically, most data comes from the northern hemisphere. Also, some latitudes (i.e., mid-latitudes [20–40°]) are more frequently sampled than other latitudinal bins, and more data from the higher and lower latitudinal bins are required to understand the spatio-latitudinal variation in drilling [10].

In terms of local ecological and environmental control on drilling, several factors like the nature of substrate, facies, water depth, water temperature, salinity, the local ecological composition of the predator and prey (i.e., tiering, size class, diversity), etc. may influence drilling predation of a particular biogeographic region. Among these factors, although the effect of temperature and salinity are relatively well-studied, the role of other biotic and abiotic factors determining large-scale predation pattern is less studied. Even the role of temperature and salinity is elusive since different contrasting results are there [8,22–25; also, Discussion]. Moreover, how different multiple confounding variables collectively control drilling predation intensity and how the collective influence of these environmental variables on predation pressure scale up over a larger biogeographic region, covering a long latitudinal extent, is controversial [13,14,26; and 10 for a detailed discussion]. As discussed by Visaggi & Kelley (2015) [8] and Mondal et al. (2019a) [10], spatio-latitudinal variation in drilling is a complex process, and there is no consensus regarding the general patterns–drilling may increase, decrease, or may remain unchanged with increasing latitude. Since ecological factors vary geographically, and it is well-known that predation pressure may be different at different biogeographies [27], a detailed study on spatio-environmental variation in predation pressure within a large biogeographic region is required. In this context, the main objective of the present work is to (1) study latitudinal and biogeographic pattern in gastropod drilling predation on molluscan prey (bivalves and gastropods) along the geomorphologically and environmentally heterogeneous eastern coast of India, covering a large latitudinal extent (c. 20° latitudes) and (2) identify how different biotic and abiotic variables determine this biogeographic pattern.

Along the eastern Indian coast, numerous ecological-biological (e.g., faunal composition, prey life mode) and environmental (e.g., substrate, energy condition) factors that influence drilling predation vary extensively. For example, along West Bengal and Odisha, coasts are dominated by mud, whereas the Tamil Nadu coast is dominated by coarse sands along with patches of seagrass habitats, and different location varies in terms of their own geomorphological, sedimentological, and faunal characteristics [28–31]. Because of this complex nature of environmental variation, the eastern Indian coast has been subdivided into multiple ecoregions [see 32,33]. Moreover, the area of the shallow shelf, which has been identified as an important variable controlling species diversity [34–37], therefore ecological interaction [3, also 38], also varies along our studied region [39]. In contrast to such ecological and environmental variability, mean temperature and salinity do not vary too much [http://www.science.oregonstate.edu/; 28] along the eastern Indian coast. However, it has been shown from the same studied region that, even this small amount of spatial variation in mean salinity can influence molluscan diversity, and the universal effect of temperature and salinity on molluscan diversity is well established [39 and references therein]. Even when temperature and salinity do not vary too much, variation in the other biotic and abiotic variables could lead to significant variation in predation. For this reason, along with the other biotic-abiotic variables, we have also included temperature and salinity in our study to test how different local ecological and environmental conditions influence drilling predation at a small spatio-temporal scale, and how these patterns scale up when performed across a large biogeographic extent, covering several latitudinal bins.

## Materials and methods

### Fieldwork, sampling, and initial processing

**Fig 1:**
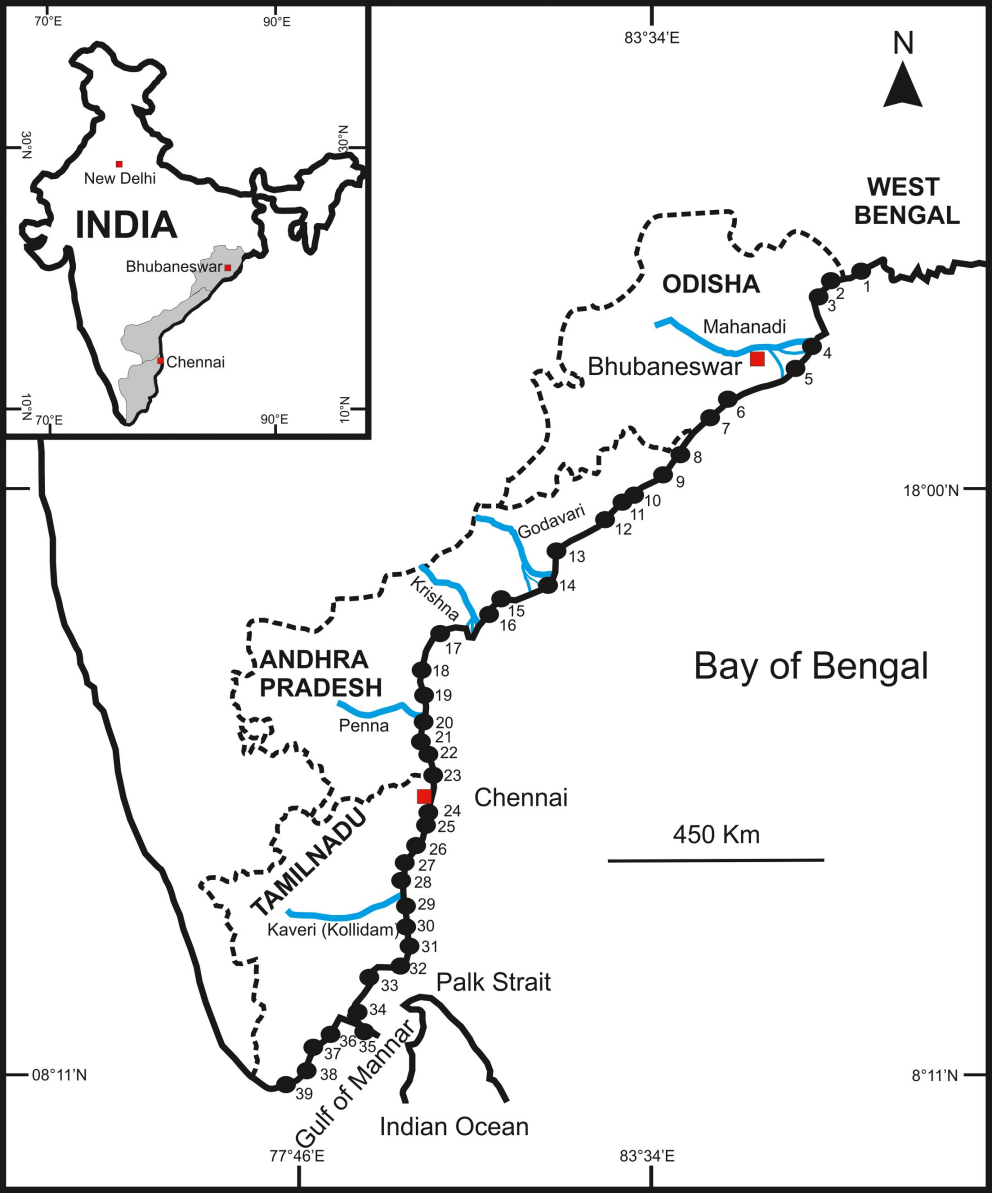
Regional map showing all sampling locations. For details about location numbers, see S1 Appendix. Inset = map of India.

76 samples are collected from 39 locations along the eastern coast of India during several field works between 2016–2018, covering about 2500 km and 15° latitudes (S1 Appendix; Fig 1). All samples have been collected from the foreshore region of the coastal beaches, sometimes open and sometimes associated with tidal flats or seagrass environments. Shell concentrations at these locations vary from moderate to high, and for this reason we decided to follow the quadrant method [8,13] and collected all specimens from the upper 5cm sediments; this procedure is, therefore, equivalent to grid-bulk sampling, i.e., bulk samples collected from a grid. All sampling stations are located reasonably away from local villages and industries to minimize anthropogenic effects.

Collected samples were brought to the laboratory and were sieved using 2mm sieve (ASTM 10). Only complete or nearly complete shells (i.e., >85% of the valves/shells intact) of molluscs (bivalves and gastropods), larger than 2mm, were used. The specimens were sorted and identified at the species level by using several books published by the Zoological Survey of India (ZSI) [29,30]. By doing so, species-level richness and abundance data of bivalve and gastropod prey were gathered for each bulk. Ecological information (i.e., prey life mode in relation to the substrate) was gathered by using the first principles of functional morphology, supplemented by others [40 and Fossilworks (www.fossilworks.com)].

### Local environmental factors

Data of different environmental variables known to affect drilling predation locally were gathered for each location and the quantitative variables [data from 39] are converted to categorical variables. Following that, all locations are subdivided into: (1) four salinity categories (unit less): 0 = low (up to 30), 1 = moderate (>30–32), 2 = high (>32–34), and 3 = very high (>34); (2) two temperature categories (°C): 0 = low (<28), 1 = moderate (28 and higher); (3) four substrate subcategories based on the prevalence of the nature of sediments: 0 = sandy (S), 1 = Muddy-sandy (MS), 2 = Sandy-rocky (SR), and 3 = Seagrass (SG); and (4) multiple ecoregions [see 32,33]: 0 for North Bay of Bengal (NBoB), 1 for North Eastern India (NEI), 2 for Central Eastern India (CEI), and 3 for South Eastern India (SEI). Along the eastern Indian coast, NEI includes the West Bengal coast characterized by high turbid conditions; NBoB includes the Odisha coast; CEI and SEI mainly includes the coastal part of Andhra Pradesh and Tamil Nadu, respectively. Both NEI and NBoB are characterized by a high rate of sedimentation and deltaic system and are geographically small. In comparison, both CEI and SEI are geographically large, covering 3–7° latitudes, and have a low rate of sedimentation. All these ecoregions are separated by ‘soft’ environmental barriers including temperature and salinity differences, sedimentological differences, among many others; the only ‘hard’ barrier is the Pamban Pass [32].

### Predation data analyses

For each sample, the total number of undrilled and completely drilled shells were counted. Drilling intensities (DI) was calculated as, DI = ∑D/∑N, where D = total number of completely drilled specimens, N = number of individuals (N = [LV+RV]/2+A, where LV = left valve, RV = right valve, A = articulated in case of bivalves; for gastropods, N = total number of gastropods present) [4]. For each location, DI was measured at three taxonomic levels: assemblage level (Assemblage Drilling Intensity, ADI), family level, and lower taxonomic (here genera) level. However, since all genera were not present in all bulks, and all families were not abundant in all locations, DI of eight most abundant bivalves and three most abundant gastropod genera (i.e., represented by at least 20 individuals collected from at least one-third [i.e., n = 12] of all studied locations) were calculated as the representative group. Similarly, 11 abundant families were considered. Along with complete drillholes, the total number of specimens with incomplete (INC), multiple (MULT), and edge (ED) [41] holes were also counted to calculate respective intensities.

Based on these above-mentioned data, the following analyses were performed: (1) assemblage drilling intensities of four tiering groups–shallow infaunal, semi-infaunal, epifaunal, and deep infaunal–were compared; (2) ADI values were compared with the corresponding prey diversity and abundance, by using Spearman correlations; (3) to identify how prey size affects drilling predation, specimens were binned into several 2cm size classes based on length (for bivalves) or height (for gastropods), and their respective DIs were compared; and, (4) drilling intensities were compared among different substrate subcategories mentioned above. In the studied regions, temperature and salinity did not vary too much, and for this reason, these variables were not compared separately with the ADI values. Since the sampling locations cover about 15 one-degree latitudinal bins and represent at least three ecoregions, drilling data were analyzed to identify the following large-scale patterns: (1) latitudinal drilling gastropod predation gradients (henceforth, LDPG) along the eastern coast of India. The correlation between latitudes and ADI was studied using Spearman correlation; (2) how INC, MULT, and ED varied with latitudes; and (3) spatial variation in ADI among different ecoregions. Kruskal-Wallis test (henceforth, KW test) and Wilcoxon test were used whenever required. All statistical tests were performed using alpha as 0.05 using R studio (R Core Team, 2012).

There is a potential chance that some of the taxa sampled here were preyed upon by muricids, along with naticid predators. However, in our case, it is expected that the results obtained here mostly reflect naticid drilling predation pattern because of the following evidences: (1) almost all drillholes resemble naticid-like morphology; (2) most of our sampling locations have soft substrates, favorable for naticids over muricids; and (3) most of our specimens are infaunal, i.e., the common prey of naticids [7,42; although some muricids consume infaunal prey]. However, to further eliminate the effect of non-naticid predation, the whole analyses were also performed only for the infaunal groups, following Visaggi & Kelley (2015) [8]. Furthermore, to account for the effect of naticid predation diversity, location-specific naticid generic richness data were gathered, for which the same set of ZSI publications mentioned above were consulted.

Finally, the non-metric multidimensional scaling (NMDS) was performed, using the Euclidean distance, to identify how different locations are grouped based on their predation levels. In other words, because we hypothesized that environmental variables (biotic: predator richness, prey richness, and prey abundance in the assemblage; abiotic factors: temperature, salinity, substrate, nutrient, ecoregions) control drilling predation, we used different biotic and abiotic variables as ‘species’ and ADI as the environmental predictor. For this purpose, ranges of ADI values were converted to category data: <10.00% (Low), 10–20.00% (Moderate), and >20.00% (High). Similarly, since the rest of the variables (i.e., temperature, salinity, substrate, ecoregions, and ADI) have been converted to categorical data, we have also converted predator and prey richness and prey abundance data into three sub-categories: predator generic richness (0: <10, 1: 10–20, 2: >20), prey generic richness (0: up to 20, 1: 21–40, 2: >40), prey abundance (0: up to 500, 1: 501–1200, 2: >1200). Along with it, the permutational multivariate analysis of variance (PERMANOVA) was used to test the same hypothesis. Along with using these multivariate techniques, ADI values are compared with the corresponding area of the shallow shelf [data from 39], using Pearson’s product-moment correlation test.

## Results

### Taxonomic and ecological information

The study analyzed drilling predation based on 65,885 specimens (54,372 bivalves and 11,513 gastropods) representing 105 bivalve genera within 35 families, and 81 gastropod genera within 49 families. When all locations were pooled, eight bivalves and three gastropod genera under 11 families, representing about 40.00% of the entire assemblages, dominate (S1 and S2 Appendices). Ecologically, the majority (i.e., ~90.00%) of specimens have semi- and shallow-infaunal life habits, i.e., c. 90.00% of all specimens can be considered as ‘naticid prey’. Moreover, more than 90.00% of all specimens are small (i.e., less than 2 cm).

### The nature of drilling predation

#### Assemblage-level predation intensities

ADI values are highly variable, ranging from 0–90.24% (median = 11.10%, mean = 15.59%). This high variability in predation intensity is also reflected when analyzed for 11 major families or genera (S2 and S3 Appendices). Spatial variation in ADI is not related to the number of genera (p = 0.57) or specimens (p = 0.45). Assemblage level edge drilling (median = 1.35) as well as incomplete drillhole intensity (INC) (median = 0.67%) are close to zero (S1 Appendix). Multiple drillholes are always rare, usually less than five in number. For these low intensities, we did not analyze spatio-environmental variation in MULT, ED, and INC.

#### Family- and genus-level drilling intensities

In all locations, only a few families and genera are drilled more intensely, and other were rarely attacked–the median value of the percentage of families and genera drilled at any location are 38.10% (mean = 35.97%) and 31.25% (mean = 30.25%), respectively (S2 and S3 Appendices). Among the eight abundant bivalve families, the two most frequently drilled are Glycymeridiidae (median = 45.90%, mean = 48.29%) and Lucinidae (mean = 40.48%); gastropods are less frequently drilled. This family-level selectivity is not related to abundance: although venerids are the most abundant of all families, representing about 30% of specimens, DI on it is not the highest, and although Glycymeridiidae is the highest drilled, they represent only 1.48% of all specimens. Among the most abundant bivalve and gastropod genera, *Glycymeris* (mean = 48.50%) is drilled with the highest intensity (S2 Appendix). Genus-level predator selectivity is not related to abundance: although the genus *Sunetta* represents about 9.50% of all specimens, DI on it is lower compared to others. Similarly, although *Glycymeris* represents only 1.47% of all specimens, DI on it is the highest.

#### Ecological and environmental control

**Fig 2:**
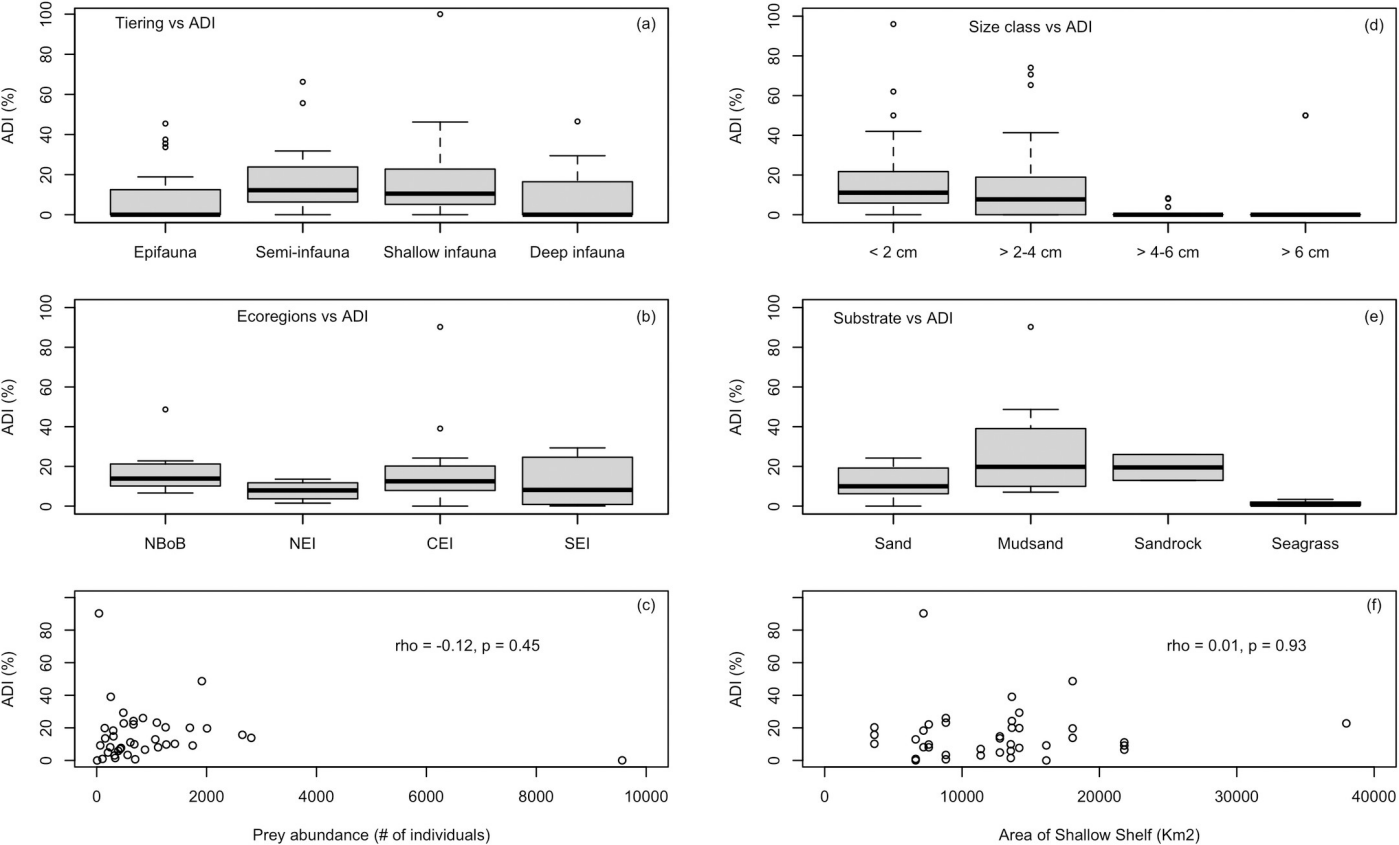
Variations of assemblage drilling intensities (ADI) on molluscs among different environmental subcategories (i.e., tiering (a), ecoregions (b), size class (d), substrate (e), prey abundance (c)) and with the area of the shallow shelf (f). Ecoregions: NBoB = Northern Bay of Bengal, NEI = North-East India, CEI = Central-Eastern India, SEI = South-Eastern India.

Comparison of drilling predation with respect to the life modes of bivalve prey reveals that different life modes have different pooled median DI values (KW test, p <0.01), median DIs being statistically higher in semi- (12.23) and shallow (10.50) infaunal forms than the rest (Fig 2). Semi- and shallow infaunal life modes are equally drilled (KW test, p = 0.29). All tier groups show high spatial variation, similar to the general pattern.

Similarly, size has a significant effect on pooled DI (KW test, df = 3, p <<0.01) (Fig 2). Pairwise comparisons reveal that, the two smallest size classes (i.e., <2cm and >2-4cm) have the highest median DI values (12.31% and 13.64%, respectively), compared to the other two (Wilcoxon test, in all cases, p << 0.01). Although the larger sizes are relatively less abundant than the smaller individuals, the relatively high abundance of these larger groups in some places (e.g., Pambali, Singarathope) did not warrant higher DI.

There is no correlation between naticid generic richness and pooled ADI (t-test, p = 0.17). Even when ADI values are compared among bins having similar naticid generic abundances, no pattern emerges (KW test, p = 0.18): bins with the least number of naticid richness (n = 9) have the highest median DI (21.22), and bins having the highest naticid richness (n = 22) have relatively low ADI (median = 9.99), although they are statistically not different (KW test, p = 0.09).

The substrate has an important control on the nature of drilling predation (KW test, p < 0.01). Muddy-sandy (median = 19.77) and Sandy-rocky (median = 19.48) environment have statistically higher pooled median DI than sandy (median = 9.99) or seagrass (median = 0.71) environments (in all cases, p < 0.01) (Fig 2). It should be noted that Sandy-rocky substrate is represented by only two locations.

### Biogeographic and latitudinal patterns

**Fig 3:**
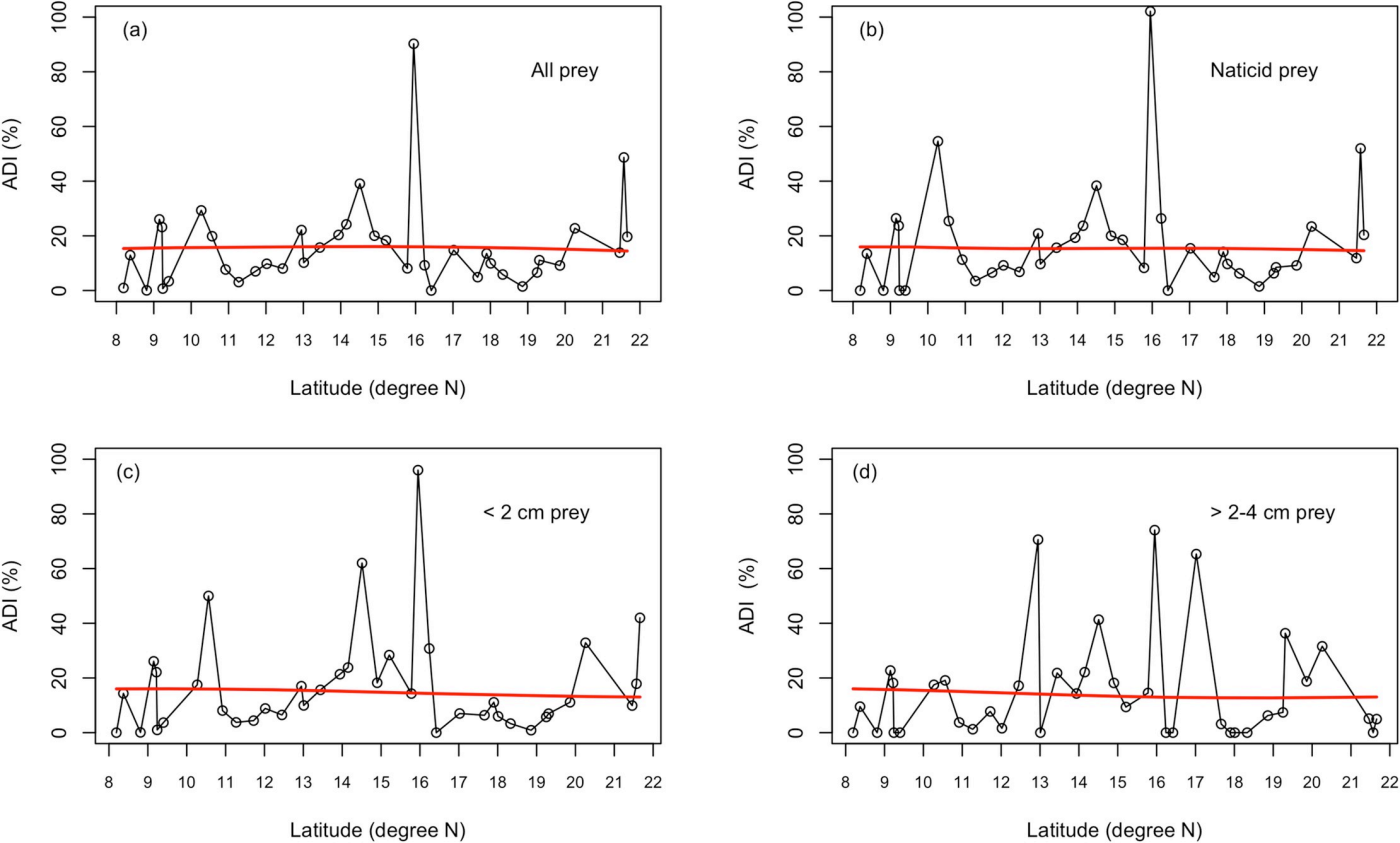
Latitudinal variation in assemblage drilling intensities (ADI) on molluscs along the eastern coast of India shows no latitudinal pattern. Note, the LOESS-fit trend line (in red) is always flat, i.e., for all prey taxa (a), only when infaunas are considered (i.e., naticid prey (b)), when two smaller size classes (c-d) are considered.

All ecoregions have statistically similar ADI values (KW test, p = 0.48), suggesting no spatial variation in predation (Fig 2). Also, there is no correlation between ADIs and latitudes (p = 0.37). A LOESS-fitted smooth curve is also flat (Fig 3). Some workers believe that using the assemblage-level drilling data could influence the likelihood of detecting any underlying latitudinal or biogeographic predation gradient, since the assemblage-level data are inherently an amalgamation of heterogeneous signals [see 43]. Therefore, we further analyzed the biogeographic pattern by looking at individual families and genera, but the general pattern remains the same for the 11 major genera and families (S2 and S3 Appendices) as well as for the shallow (p = 0.62) or semi-infaunal (p = 0.81) groups. Similarly, for the ‘naticid prey’ (more than 90.00% are <2cm in size, living infaunally in the soft substrate), no latitudinal emerged (p = 0.53). Size-class specific or substrates-specific ADI values reveal that there is no distinct latitudinal pattern in drilling intensity (Fig 3; S3 Appendix).

**Fig 4:**
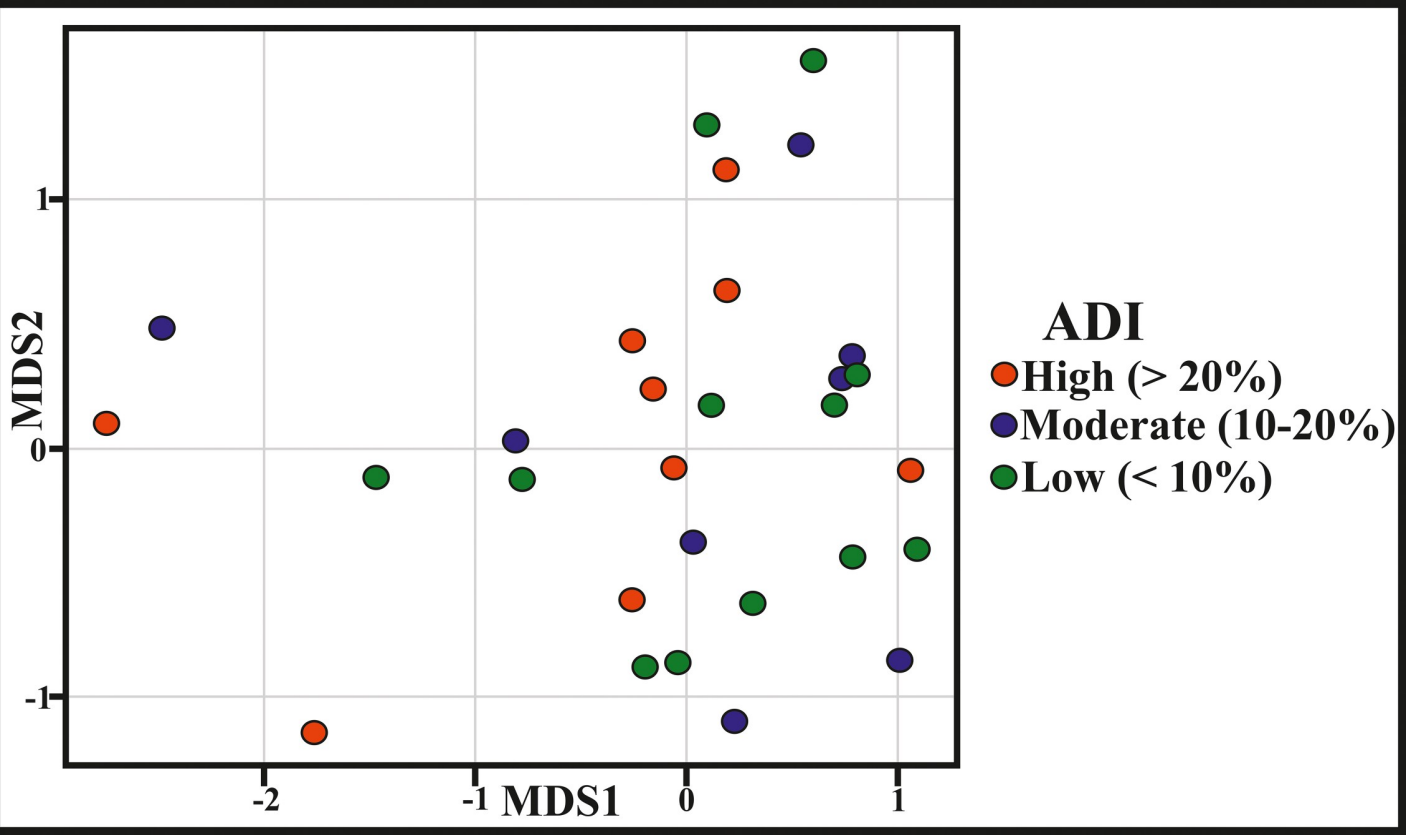
Non-metric multidimensional scaling with Euclidean distance (k = 2, stress = 0.15) of sampling sites shows poor clustering based on their respective molluscan assemblage drilling intensities (ADI), suggesting that although several locations have similar environments, they differ in their respective drilling intensities.

NMDS plot (k = 2, stress = 0.15) shows no cluster among locations with similar ADI values (Fig 4). PERMANOVA result also indicate that ADI values are not an important classifier to cluster different locations (F = 1.04, df = 2, R^2^ = 0.05, p = 0.40). Analysis of Variance (ANOVA) results also support this (F = 0.85, df = 2, p = 0.44). Therefore, similarities in the environmental factors–prey and predator generic richness, prey abundance, ecoregion identity, temperature, salinity, nutrient, and substrate–do not guarantee similarities in ADI values. Moreover, the shallow shelf area does not show any correlation with ADI values (p = 0.93) (Fig 2). Therefore, local ecological and environmental factors appear to be more important in determining the regional pattern, at least along the eastern coast of India.

## Discussion

Based on a large dataset covering a great latitudinal (15°) and biogeographic (c. 2500 km, including several ecoregions and ocean sub-basins) extent, we find extensive spatial variation in drilling predation along the eastern coast of India, which has been observed by others in their local [44] to regional [14,26,45–47] to large-scale geographic [7,8,13,22] studies as well as in fossil records [23–25,48,49]. The eastern Indian coast is ecologically, sedimentologically, and environmentally very dynamic, showing high variation in the nature of substrate, energy condition, productivity, faunal diversity and composition, and prey ecology [28–33,39], which are known to influence drilling predation [8,22–25]. Different degree of variation in these confounding variables at different locations, even when temperature and salinity do not vary too much, therefore lead to significant variation in predation. Interestingly, we find no biogeographic or latitudinal gradient in drilling predation along the eastern Indian coast. In fact, different ecoregions, having their distinctive biotic and abiotic environmental variables, show similar predation intensities, and DI varies within the same latitudinal bin, oceanic sub-basins, or even the same ecoregions. The absence of any gradient is also supported by the NMDS plot, which reveals no cluster of sampling locations based on their respective ADI values. Our results suggest that, different biotic and abiotic variables influence predator-prey interaction only locally, as discussed below.

### Robustness of the observed pattern

Taphonomic overprinting in our samples appears to be minimum for several reasons. For example, left and right valve ratios are always almost equal to 1 (median = 0.99), and the presence of articulated shells in many samples (i.e., in 15 locations) suggests minimum post-mortem transport. Also, shells are always pristine showing preserved ornamentations and external shell colors, and in many cases periostracum layers are intact, suggesting minimum biostratinomic impacts. Moreover, samples have smaller as well as larger size classes, suggesting no significant size sorting. Under these minimum biostratinomic biases, drilled shells are less likely to be preferentially removed by taphonomic agents [50–52 and discussion therein]. Moreover, in our samples, shell fragments with identifiable naticid holes were observed on rare occasions (only 20 shells, <0.05% of total bivalve specimens studied), but breaks never passing through the drill hole, suggesting that the presence of hole did not weaken the shells, at least under low biostratinomic regimes [50 and references therein]. Therefore, although it has been proposed that the drilled and undrilled valves may be transported differentially, leading to distortion of drilling intensity, we think our local ADI values as well as the observed biogeographic patterns are less likely to be affected. A recent study from the eastern Indian coast [53], analyzing dead molluscan assemblages from 16 of our 36 sampling locations, suggested that although the coastal assemblages are time-averaged, differential degree of time averaging may not be an important factor producing any geographic trend in ADI [see 53]. Also, durophagy bias–removal of shells by crushing may artificially inflate DI values [e.g., 54]–could influence ADI. However, since all studied locations are within the tropics, the intensity of shell-crushing predation are most likely affecting ADIs of all the localities almost equally. The absence of any geographic pattern in the present case further suggests that the durophagy bias alone is unlikely to be responsible to detrend any existing latitudinal drilling predation trend.

### Local biotic-abiotic control versus large-scale patterns

Temperature and salinity can be important factors in determining local predation [23–26]. However, in our samples, we observe complete decoupling of temperature and DI: although the temperature does not vary significantly, high variation in DI is observed. Also, samples located towards the lower latitude do not always have higher drilling, and samples with higher latitude may also have high DI values. Similarly, although salinity is generally high towards the south in our case, DI values do not decrease towards the south, showing no correlation between salinity and DI as well [also see 14]. The absence of any direct correlation between these variables with ADI as well as lack of any biogeographic pattern in DI happens because, in our studied region, temperature or salinity do not systematically vary along a spatial or latitudinal gradient. Moreover, in a large biogeographic region, salinity and temperature show a complex interplay between them, influencing the nature of ecological interaction [8,23]. Even for a longer time interval, the relationship is complex: while an increase in DI with elevated temperature has been observed by few [55], intervals with higher temperature do not always have higher DI values. For example, for the Jurassic-onwards, the tropics did not have the highest DI for many time bins [10].

On the other hand, the substrate can be an important abiotic factor to determine the nature of the local ecological structure, therefore ecological interaction [56,57], but the relationship is not straightforward. Although we find higher pooled DI in fine-grained substrates than the sandy beaches, as observed by few others [58], this observation is counterintuitive: naticid predators prefer sandy-muddy soft substrate, supporting its infaunal life mode. Maybe for this, the relationship can vary with taxonomy or biogeography [23,24]. Even more, Hansen & Kelley (1995) [22], having dealt with much larger samples, did not find any substrate-controlled pattern in predation. Therefore, local factors could be more important in influencing large spatio-temporal patterns.

The main local biotic controls include prey morphology (e.g., shell shape), and ecology (i.e., life mode). In our case, even we consider any specific prey family or genera, having similar prey morphology and life mode, no gradient in predation is evident. Similarly, while analyzing the predation gradient by considering these infaunas only, we did not find a latitudinal trend, suggesting that the effect of prey life mode on drilling is local. Strong preference for shallow and semi-infaunal prey in our case, as also observed by others [42] can be attributed to two potential causes: since the majority of our sampling locations have soft substrates, and naticid gastropods preferentially attack infaunal prey over their epifaunal counterpart, the chances of getting traces of naticid predation is high. This is also supported by the fact that the majority of our shells carry naticid-like holes. This idea suggests that the nature of substrate can determine the nature of prey and predator composition, including life mode and taxonomic compositions at any location, influencing local drilling predation [8].

The available prey size classes may also have a local effect in determining local drilling predation intensities. Since naticids maintain strict size selectivity while drilling their molluscan prey [23,25,26,44; but see 24], smaller specimens are more frequently drilled in all locations, showing a preference for smaller prey size. Although this is evident in all samples, no biogeographic or latitudinal pattern is observed at all. This suggests, the local ecological patterns do not scale up to long-term temporal patterns: an increase in predator-prey size from the Cambrian onwards [9] does not warrant the synchronous increase in predation intensity from the Cambrian [see 2].

Local predation intensity may be controlled by the predator and prey diversity and their taxonomic identities [22,26]. Although different naticid species are present in India, three genera, *Natica*, *Polinices*, and *Sinum* are the most abundant throughout the entire eastern coast. Other than these, *Tectonatica*, *Naticarius*, *Eunaticina*, *Tanea*, *Neverita*, *Mammilla*, and *Glossaulax* are also present [29,30], but less in abundance and richness. It is possible that the presence of different naticid predators at different locations could influence the observed results, but the absence of any biogeographic pattern in DIs suggests that spatial difference in predator types is not a potential cause in our case. Moreover, the three most abundant genera—*Natica*, *Polinices*, and *Sinum–*are present throughout the eastern coast, although spatially DI varies significantly. Similarly, there is no direct correlation between naticid generic richness and respective ADI values, as suggested by other authors [8,10,13,24,55]. Even within the same prey types, no latitudinal or biogeographic pattern emerges [S2 and S4 Appendices] and there is no correlation between prey abundance and ADI values. All these suggest that the absence of any pattern cannot be attributed to predator-prey diversities and abundances. The lack of any pattern could be explained by the fact that predators always prefer specific prey, showing strong taxon selectivity (see below). Furthermore, the large-scale patterns may not be extrapolated to small-scale studies, and small-scale patterns do not always scale up to the regional, large-scale pattern.

### What matters the most

One regional pattern is prevalent in our study: some taxa are consistently more frequently drilled than others, irrespective of their relative abundances locally as well as biogeographically, showing strong ‘taxon selectivity’. In our case, we identified at least five taxa that are drilled at least with 15.00% intensity. There is a strong, positive correlation between the proportional abundance of these preferred prey and the respective assemblage drilling intensity (Spearman rho = 0.59, p <<0.01), suggesting that the presence of preferred prey determines the local ecological structure as well as the intensity of predation at each location. These families are more preferred because of their sluggish motility, tiering characteristics, overall adult shell sizes, and the nature of weak antipredatory defenses against drilling [7,15,22,56,59, and many others].

However, it should be noted that different prey is preferred at different locations, suggesting a complex nature of prey preference for naticids. While the most preferred prey are not commonly available at any location, predators switch (prey switching) towards ‘preferred alternative prey’: as shown by Alexander & Dietl (2001) [26], towards the northern part of the Atlantic coast of the USA, naticid predators do not prefer small-sized species, *Donax variabilis*, although they are extremely abundant. However, near the south, smaller abundant prey like *Divalinga quadrisulcata* are frequently drilled, although they are also smaller in size, simply because other preferred prey are less abundant. This type of prey switching, leading to change in DI, has also been shown experimentally [60].

Moreover, the median proportional abundance of these preferred prey is higher in muddy-sandy (41.39% of total fauna at any location) than sandy (17.15%) and seagrass (0.27%) locations (KW test, p <0.01). Mainly for this reason, muddy-sandy locations have higher ADI than other substrates in our case. This indicates that substrate is the most important factor to determine the presence of preferred prey in our locations. All these, together, indicate that substrate determines the nature of taxonomic and ecological compositions, and along with it, the taxonomic identities (i.e., the preferred prey items) determine the local ADIs. Lack of variation in abundance of preferred prey or the nature of substrate along the spatio-latitudinal gradient of the eastern India coast results in lack of any large-scale latitudinal or biogeographic pattern.

## Biogeographic and latitudinal variation in drilling predation (through time)

LDPG is complex, which is becoming more and more evident with new studies–some reported an increase in DI with latitude [6,8,26,46,61,62], whereas, others reported inverse or a much more mixed relationship [12,13,47]. In fact, a mixed pattern is much more common when we pool all these data together and data are categorized into their respective biogeographic regions of the world: within the Indian subcontinents, drilling may increase towards the mid-latitudes [46] or may show no pattern at all [47]. Similarly, for the Americas also, the pattern is mixed: drilling may increase [8,26] or decrease [12] towards the tropics. This suggests that latitude-controlled predation gradient may in fact be mixed and is strongly dependent upon the study groups, or biogeographic and latitudinal coverage [see 8]. In the fossil records also, similar complex patterns emerge when Mondal et al. (2019a) [10] recently analyzed the latitudinal pattern of drilling predation from the Cretaceous up to the Pleistocene. The study revealed that, the latitudinal pattern of gastropod drilling predation evolves with time, with evolving biotic and abiotic factors that control predation [see 1].

The degree of spatial variation in DI within a time bin may sometimes be much greater than temporal variations observed between two consecutive time bins [2,3]. This pattern is so prevalent that it can be seen for single species as well as for the entire family or the assemblage [6,23]. For example, Goswami et al. (2020) [49] reported that, temporally between two formations, median DI ranges from 3.76–5.41% (i.e., varies only about 1.75%), whereas, within each formation, DI varies by as much as 6–8.00%. Chattopadhyay et al. (2016) [25] also found a large among-locality variation (c. 25.00%), which was greater than the variation between two stratigraphic units. The same pattern was also observed by Kelley & Hansen (2006) [55] for the entire Cenozoic. As a consequence, interpretation of temporal change in DI should be treated with cautions and the context of spatial variation within each time should be accounted for [13]. Since spatio-environmental variations, acting as local, short-term “noises” [12], could potentially overcast the large-scale biogeographic patterns, as in our case, no large-scale pattern will emerge, even if sampling is complete or the methodology is strong. Fortunately, people have found several distinct, long-term patterns emerging from the studied of fossil records, suggesting that, while considering all spatially-ranged samples within a time bin, spatio-latitudinal variation ‘hopefully average out’ and the reminiscent large-scale pattern remains emergent [23].

## Conclusion

The nature of spatio-environmental and latitudinal variation in gastropod drilling predation across the eastern Indian coast—covering about 2500 km and including about 15° latitudes, incorporating several ecoregions and ocean basins—were studied. We find no latitudinal or biogeographic pattern in drilling, and ADI values show high latitudinal or biogeographic variability. Several biotic and abiotic factors known to influence drilling predation only have local effects. For example, small infaunal prey, living in sandy and muddy substrates are always drilled more frequently than others, suggesting that the nature of substrate determines the taxonomic and ecological compositions of the local prey compositions, and together with different biotic and abiotic factors determine the local predation intensity.

## Supporting information

S1 Appendix Environmental details and the respective predation intensity data of the studied locations. For additional details of exact values of salinity, see text. ADI = assemblage drilling intensity; #INC and # MULT = total number of incomplete and multiple drilled specimens, respectively; ED = total number of edge drilled specimens; n = number of individuals; Ecoregions: NBoB = North Bay of Bengal, NEI = North Eastern India, CEI = Central Eastern India, SEI = South Eastern India; S = Sandy, MS = Muddy-sandy, SR = Sandy-rocky, SG = Seagrass.
(DOCX)

S2 Appendix Drilling intensities (%) of the most abundant genera for all locations. For each location, the proportion of total genera drilled is also provided. + = the genus is represented by less than 10 individuals, therefore corresponding DI values are not calculated.— = absent.
(DOCX)

S3 Appendix Substrates-specific ADI values along the entire studied region. The LOESS-fit trend line (in red) is flat for both types of substrate-specific analyses (top figure: Sandy substrate, bottom figure: Muddy-sandy substrate), indicating the absence of any latitudinal pattern. Numbers along the x axes represent the studied locations arranged according to decreasing latitudes form left to right. Number-specific locations names can be found in S1 Appendix.
(DOCX)

S4 Appendix Drilling intensities (%) of the most abundant families for all locations. For each location, the proportion of total families drilled is also provided. + = the family is represented by less than 10 individuals, therefore corresponding DI values are not calculated.— = absent. Values within brackets right after taxa names indicate their abundances in the entire collection.
(DOCX)
