# Supplementary material for: High biogeographic and latitudinal variability in gastropod drilling predation on molluscs along the eastern Indian coast: Implications on the history of fossil record of drillholes
Source: PLoS One. 2021 Aug 26;16(8):e0256685. doi: 10.1371/journal.pone.0256685 (PMC8389373; doi:10.1371/journal.pone.0256685)
Supplement: S1 Appendix — For additional details of exact values of salinity, see text. ADI = assemblage drilling intensity; #INC and # MULT = total number of incomplete and multiple drilled specimens, respectively; ED = total number of edge drilled specimens; n = number of individuals; Ecoregions: NBoB = North Bay of Bengal, NEI = North Eastern India, CEI = Central Eastern India, SEI = South Eastern India; S = Sandy, MS = Muddy-sandy, SR = Sandy-rocky, SG = Seagrass. (DOCX) [file pone.0256685.s001.docx]

**APPENDIX S1** Environmental details and the respective predation intensity data of the studied locations. For additional details of exact values of salinity, see text. ADI = assemblage drilling intensity; #INC and # MULT = total number of incomplete and multiple drilled specimens, respectively; ED = total number of edge drilled specimens; n = number of individuals; Ecoregions: NBoB = North Bay of Bengal, NEI = North Eastern India, CEI = Central Eastern India, SEI = South Eastern India; S = Sandy, MS = Muddy-sandy, SR = Sandy-rocky, SG = Seagrass.

| No. | Locations | Latitude (N) | Ecoregion | Salinity | Substrate | ADI (%) | #INC | #MULT | #ED | n |
| --- | --- | --- | --- | --- | --- | --- | --- | --- | --- | --- |
| 1 | Tajpur | 21.66 | NBoB | Low | MS | 19.68 | 2 | 0 | 0 | 2007.5 |
| 2 | Dogra | 21.57 | NBoB | Low | MS | 48.67 | 1 | 1 | 0 | 1913.0 |
| 3 | Chandipur | 21.46 | NBoB | Low | MS | 13.86 | 11 | 0 | 0 | 2813.0 |
| 4 | Paradeep | 20.26 | NBoB | Low | S | 22.76 | 1 | 1 | 12 | 492.0 |
| 5 | Chandrabhaga | 19.86 | NBoB | Low | S | 9.16 | 0 | 2 | 14 | 1746.5 |
| 6 | Arjipalli | 19.31 | NBoB | Low | S | 11.10 | 0 | 0 | 2 | 612.5 |
| 7 | Gopalpur | 19.25 | NBoB | Low | S | 6.60 | 2 | 0 | 9 | 878.5 |
| 8 | Yekuvuru | 18.86 | NEI | Moderate | S | 1.49 | 0 | 0 | 1 | 336.5 |
| 9 | Kalingapatnam | 18.33 | NEI | Moderate | S | 5.86 | 0 | 0 | 5 | 392.5 |
| 10 | Konada | 18.01 | NEI | Moderate | MS | 9.93 | 0 | 0 | 3 | 684.5 |
| 11 | Bheemunipatnam | 17.90 | NEI | Moderate | S | 13.55 | 0 | 0 | 6 | 155.0 |
| 12 | Yarada | 17.66 | CEI | Moderate | S | 4.88 | 2 | 0 | 4 | 205.0 |
| 13 | Polavarem | 17.02 | CEI | Moderate | S | 14.78 | 3 | 0 | 3 | 304.5 |
| 14 | Odalarevu | 16.42 | CEI | Moderate | S | 0.00 | 0 | 0 | 0 | 8.5 |
| 15 | Manginipudi | 16.24 | CEI | Moderate | MS | 9.23 | 0 | 0 | 0 | 65.0 |
| 16 | Koduru | 15.95 | CEI | Moderate | MS | 90.24 | 0 | 0 | 0 | 41.0 |
| 17 | Ramapuram | 15.78 | CEI | Moderate | S | 8.11 | 1 | 0 | 0 | 246.5 |
| 18 | Binginapalli | 15.21 | CEI | Moderate | S | 18.33 | 0 | 0 | 3 | 300.0 |
| 19 | Thummalapentha | 14.90 | CEI | Moderate | S | 20.05 | 7 | 0 | 30 | 1695.5 |
| 20 | Mypadu | 14.51 | CEI | Moderate | MS | 39.06 | 1 | 0 | 11 | 256 |
| 21 | Srinivasa Sathravam | 14.15 | CEI | Moderate | S | 24.20 | 1 | 1 | 3 | 669.5 |
| 22 | Pambali | 13.94 | CEI | High | S | 20.33 | 3 | 0 | 7 | 1254 |
| 23 | Pulicat | 13.44 | CEI | High | S | 15.73 | 3 | 1 | 6 | 2651 |
| 24 | Broken Bridge | 13.01 | CEI | High | S | 10.19 | 5 | 1 | 13 | 1422.5 |
| 25 | Neelangarai | 12.95 | CEI | High | S | 22.12 | 1 | 1 | 2 | 669 |
| 26 | Vayalur | 12.45 | CEI | High | S | 8.06 | 4 | 0 | 6 | 1117 |
| 27 | Pondicherry | 12.02 | CEI | High | S | 9.79 | 1 | 0 | 3 | 1266 |
| 28 | Singarathope | 11.72 | CEI | High | MS | 7.03 | 1 | 0 | 2 | 426.5 |
| 29 | Thirumullaivasal | 11.27 | CEI | High | S | 3.08 | 0 | 1 | 0 | 325 |
| 30 | Karaikal | 10.92 | CEI | High | S | 7.69 | 0 | 0 | 0 | 442 |
| 31 | VKP Beach | 10.56 | CEI | High | MS | 19.86 | 0 | 0 | 0 | 146 |
| 32 | Kodiyakarai | 10.27 | SEI | High | MS | 29.31 | 0 | 0 | 0 | 484.5 |
| 33 | Gopalpattinam | 9.24 | SEI | High | SG | 3.38 | 0 | 0 | 0 | 562 |
| 34 | Athiyuthur | 9.40 | SEI | High | SG | 0.71 | 1 | 0 | 0 | 700.5 |
| 35 | Dhanushkodi | 9.22 | SEI | High | S | 23.21 | 5 | 1 | 7 | 1094.5 |
| 36 | Alavangulam | 9.15 | SEI | High | SR | 26.02 | 3 | 0 | 8 | 841.5 |
| 37 | Muthunagar | 8.81 | SEI | Very High | SG | 0.06 | 0 | 0 | 0 | 9563 |
| 38 | Manapadu | 8.37 | SEI | Very High | SR | 12.94 | 1 | 0 | 9 | 1066.5 |
| 39 | Avudaiyalpuram | 8.19 | SEI | Very High | S | 0.95 | 2 | 0 | 0 | 105 |
