# Supplementary material for: High biogeographic and latitudinal variability in gastropod drilling predation on molluscs along the eastern Indian coast: Implications on the history of fossil record of drillholes
Source: PLoS One. 2021 Aug 26;16(8):e0256685. doi: 10.1371/journal.pone.0256685 (PMC8389373; doi:10.1371/journal.pone.0256685)
Supplement: S2 Appendix — For each location, the proportion of total genera drilled is also provided. + = the genus is represented by less than 10 individuals, therefore corresponding DI values are not calculated.— = absent. (DOCX) [file pone.0256685.s002.docx]

**APPENDIX S2** Drilling intensities (%) of the most abundant genera for all locations. For each location, proportion of total genera drilled is also provided. + = the genus is represented by less than 10 individuals, therefore corresponding DI values not calculated. - = absent.

| Location | *Timoclea* (17.01%) | *Meretrix* (2.93%) | *Sunetta* (9.49%) | *Donax* (2.40%) | *Glycymeris* (1.47%) | *Mactra* (1.89%) | *Anadara* (1.01%) | *Tellina* (0.61%) | *Turritella* (0.57%) | *Cerithidea* (0.78%) | *Umbonium* (1.12%) | Proportion of genera drilled (%) |
| --- | --- | --- | --- | --- | --- | --- | --- | --- | --- | --- | --- | --- |
| Tajpur | 19.94 | + | - | 34.67 | - | 39.34 | + | - | - | - | 10.71 | 40.74 |
| Dogra | 55.67 | + | + | 28.57 | - | 6.90 | - | - | - | - | 5.56 | 50.00 |
| Chandipur | 9.58 | - | + | 52.94 | + | 19.30 | + | 48.19 | + | 6.90 | - | 44.44 |
| Paradeep | + | 4.35 | 20.00 | 31.03 | + | - | + | + | + | - | - | 47.83 |
| Chandrabhaga | 45.45 | 11.40 | 8.63 | 2.21 | + | + | + | + | + | - | - | 65.22 |
| Arjipalli | 10.96 | + | 19.51 | 0.00 | 17.39 | 0.00 | + | 0.00 | + | 23.93 | + | 27.87 |
| Gopalpur | + | 4.82 | 3.83 | + | 48.00 | + | + | + | + | - | - | 29.63 |
| Yekuvuru | - | + | 1.60 | + | + | - | + | - | + | - | - | 8.33 |
| Kalingapatnam | - | + | 3.47 | + | + | - | 14.29 | - | - | - | - | 20.00 |
| Konada Mohona | + | 6.84 | 3.21 | + | 57.58 | + | + | + | - | - | - | 29.63 |
| Bheemunipatnam | + | 15.20 | 2.94 | + | 45.16 | + | + | + | - | - | - | 11.53 |
| Yarada | + | 0.99 | 3.28 | + | - | + | + | - | + | + | + | 38.10 |
| Polavarem | + | 0.00 | 17.84 | 0.00 | - | - | + | - | - | - | - | 16.67 |
| Odalarevu | - | + | - | - | - | - | - | - | - | - | - | 0.00 |
| Manginipudi | - | + | - | - | - | + | 31.58 | - | + | + | - | 4.76 |
| Koduru | - | - | - | + | - | - | 100.00 | - | - | - | - | 25.00 |
| Ramapuram | + | 0.00 | + | + | - | + | 59.09 | - | - | - | + | 25.00 |
| Binginapalli | 52.75 | 7.89 | 7.84 | 0.00 | + | 36.36 | 10.26 | + | - | - | - | 34.21 |
| Thummalapentha | 22.05 | 6.12 | 19.19 | 4.08 | 46.15 | 12.8 | 13.11 | 15.38 | - | - | 4.55 | 40.00 |
| Mypadu | 56.25 | + | 55.38 | + | 86.49 | 48.48 | 17.65 | - | - | - | - | 36.36 |
| Srinivasa Sathravam | 39.66 | 0.00 | 17.14 | 3.47 | 51.02 | 46.96 | 12.05 | 17.65 | - | - | - | 34.88 |
| Pambali | 33.26 | + | 27.39 | 0.00 | 50.59 | 10.64 | 8.59 | 21.88 | - | - | - | 37.21 |
| Pulicat | 20.73 | 5.26 | 7.35 | 2.40 | 40.51 | + | 18.60 | 8.64 | + | - | - | 62.96 |
| Broken Bridge | 18.38 | 4.63 | 2.41 | 0.69 | 90.91 | + | + | 0.00 | + | - | - | 44.74 |
| Neelangarai | 29.89 | 4.44 | 0.00 | 0.00 | + | + | + | + | 9.09 | - | - | 40.00 |
| Vayalur | 12.87 | 5.41 | 1.78 | 2.00 | + | + | 3.57 | + | 0.00 | 36.36 | + | 42.22 |
| Pondicherry | 15.08 | 1.12 | 3.34 | + | + | 0.00 | 0.00 | + | + | - | - | 25.81 |
| Singarathope | 14.29 | 9.84 | 3.39 | 0.00 | + | 6.90 | 7.79 | + | + | 0.00 | 0.00 | 29.27 |
| Thirumullaivasal | 18.6 | 0.00 | 2.20 | 0.00 | - | + | 0.00 | + | 1.75 | - | 0.00 | 24.14 |
| Karaikal | 16.16 | 0.00 | + | + | - | - | 7.25 | 22.22 | 2.27 | - | + | 29.73 |
| VKP Beach | + | + | - | + | - | - | 22.58 | - | + | - | - | 41.18 |
| Kodiyakarai | 80.00 | + | - | 0.00 | - | 30.99 | 63.94 | + | 5.15 | - | + | 32.61 |
| GPM | - | - | - | + | - | + | + | - | - | - | 0.00 | 8.70 |
| Athiyuthur | 0.00 | 8.70 | - | 0.00 | - | - | - | + | - | - | 0.40 | 16.67 |
| Dhanushkodi | 31.92 | + | 21.96 | 3.85 | 42.70 | + | - | 16.95 | + | - | + | 37.50 |
| Alavangulam | 41.26 | 5.39 | 43.04 | 0.00 | 22.56 | + | + | + | + | - | - | 45.24 |
| Muthu Nagar | + | + | - | 0.00 | + | + | + | - | - | 0.00 | 0.98 | 6.82 |
| Manapadu | 12.49 | - | + | + | 31.48 | + | + | - | - | - | - |  |
| Avudaiyalpuram | - | + | - | - | + | - | - | - | + | - | + |  |
