# Supplementary material for: High biogeographic and latitudinal variability in gastropod drilling predation on molluscs along the eastern Indian coast: Implications on the history of fossil record of drillholes
Source: PLoS One. 2021 Aug 26;16(8):e0256685. doi: 10.1371/journal.pone.0256685 (PMC8389373; doi:10.1371/journal.pone.0256685)

**APPENDIX S3** Substrates-specific ADI values along the entire studied region. The LOESS-fit trend line (in red) is flat for both types of substrate-specific analyses (top figure: sandy substrate, bottom figure: muddy-sandy substrate), indicating the absence of any latitudinal pattern. Numbers along the x axes represent the studied locations arranged according to decreasing latitudes form left to right. Number-specific locations names can be found in Appendix S1.


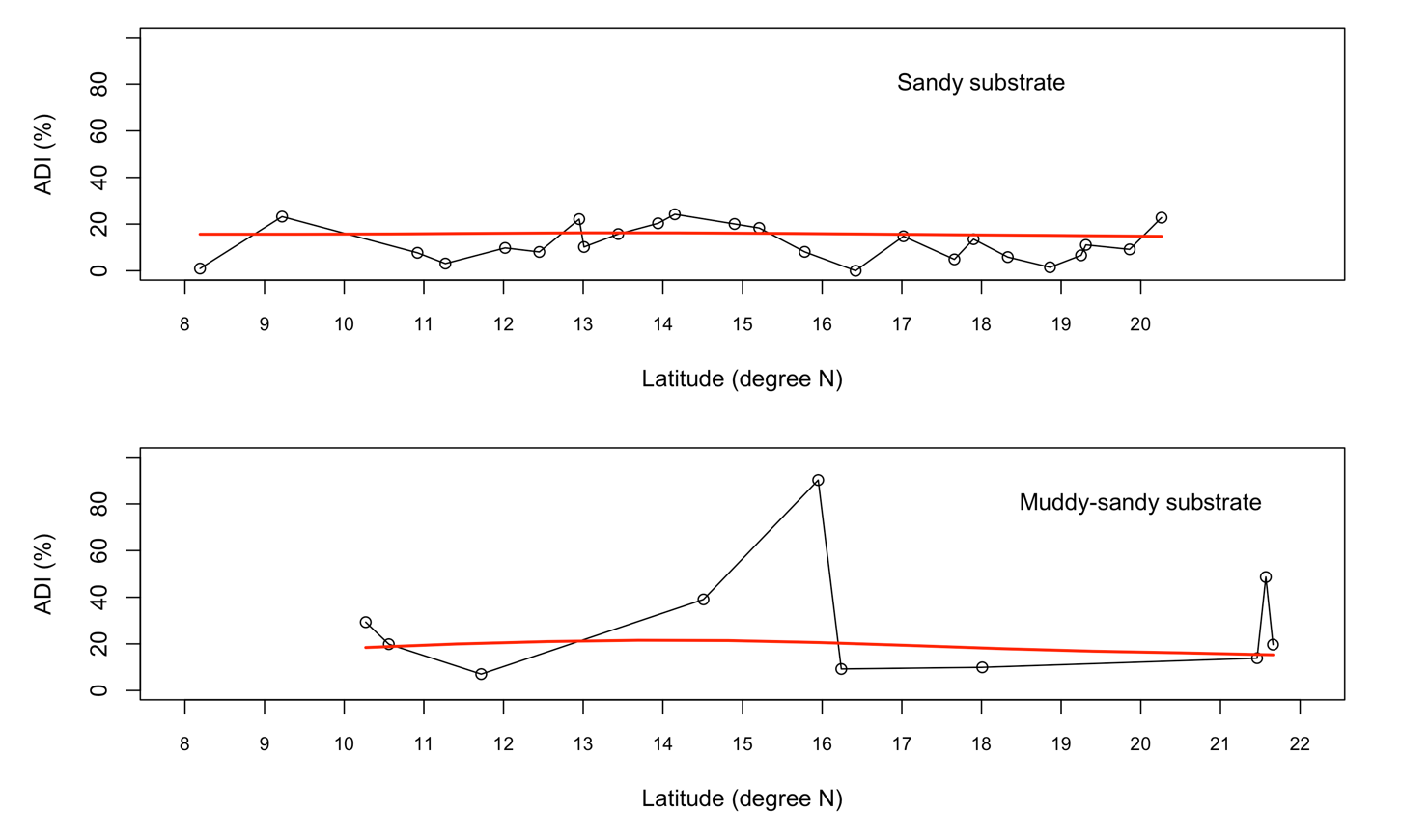

Supplement: S3 Appendix — The LOESS-fit trend line (in red) is flat for both types of substrate-specific analyses (top figure: Sandy substrate, bottom figure: Muddy-sandy substrate), indicating the absence of any latitudinal pattern. Numbers along the x axes represent the studied locations arranged according to decreasing latitudes form left to right. Number-specific locations names can be found in S1 Appendix. (DOCX) [file pone.0256685.s003.docx]
