# Supplementary material for: High biogeographic and latitudinal variability in gastropod drilling predation on molluscs along the eastern Indian coast: Implications on the history of fossil record of drillholes
Source: PLoS One. 2021 Aug 26;16(8):e0256685. doi: 10.1371/journal.pone.0256685 (PMC8389373; doi:10.1371/journal.pone.0256685)
Supplement: S4 Appendix — For each location, the proportion of total families drilled is also provided. + = the family is represented by less than 10 individuals, therefore corresponding DI values are not calculated.— = absent. Values within brackets right after taxa names indicate their abundances in the entire collection. (DOCX) [file pone.0256685.s004.docx]

**APPENDIX S4** Drilling intensities (%) of the most abundant families for all locations. For each location, proportion of total families drilled is also provided. + = the family is represented by less than 10 individuals, therefore corresponding DI values not calculated. - = absent. Values within brackets right after taxa names indicate their abundances in the entire collection.

| Locations | Carditidae (0.42%) | Lucinidae (0.21%) | Veneridae (30.28%) | Arcidae (2.47%) | Cardiidae (0.30%) | Glycymerididae (1.48%) | Tellinidae (0.83%) | Mactridae (1.91%) | Muricidae (0.10%) | Turritellidae (0.60%) | Naticidae (0.56%) | Proportions of families drilled (%) |
| --- | --- | --- | --- | --- | --- | --- | --- | --- | --- | --- | --- | --- |
| Tajpur | - | - | 19.99 | + | - | - | + | 38.71 | + | - | 8.57 | 41.18 |
| Dogra | - | - | 55.56 | - | - | - | + | 6.90 | - | - | 26.32 | 83.33 |
| Chandipur | - | - | 9.81 | + | - | + | 47.06 | 19.3 | 0.00 | + | 28.61 | 45.00 |
| Paradeep | + | + | 20.28 | + | - | + | + | - | - | + | - | 53.33 |
| Chandrabhaga | + | + | 9.04 | 28.57 | - | + | + | + | - | + | - | 80.00 |
| Arjipalli | + | 5.26 | 12.41 | 17.82 | + | 17.39 | 0.00 | 0.00 | + | + | 11.11 | 33.33 |
| Gopalpur | + | 34.15 | 4.28 | + | + | 48.00 | + | + | - | + | - | 38.46 |
| Yekuvuru | - | + | 1.54 | + | + | + | - | - | - | + | - | 10.00 |
| Kalingapatnam | - | - | 3.69 | 13.79 | + | + | + | - | + | - | - | 38.46 |
| Konada | + | + | 4.28 | + | + | 57.58 | + | + | - | - | - | 33.33 |
| Bheemunipatnam | - | + | 11.20 | + | - | 45.16 | + | + | - | - | - | 16.67 |
| Yarada | - | + | 2.93 | + | + | - | - | + | - | + | - | 38.46 |
| Polavarem | - | - | 15.81 | + | - | - | + | - | - | - | - | 25.00 |
| Odalarevu | - | - | + | - | - | - | - | - | + | - | - | 0.00 |
| Manginipudi | - | - | + | 30.77 | - | - | - | + | + | - | + | 7.14 |
| Koduru | - | - | + | 104.35 | - | - | - | - | - | - | - | 40.00 |
| Ramapuram | - | - | 3.08 | 8.37 | + | - | + | + | - | - | - | 30.00 |
| Binginapalli | 4.55 | - | 19.28 | 11.76 | + | + | + | 36.36 | - | + | - | 38.10 |
| Thummalapentha | 28.57 | + | 21.73 | 11.97 | 9.52 | 45.90 | 13.64 | 12.80 | - | + | - | 48.28 |
| Mypadu | 18.46 | - | 64.44 | 13.59 | 0.00 | 84.21 | + | 45.71 | - | 0.00 | - | 35.29 |
| Srinivasa Sathravam | 19.28 | + | 31.68 | 8.05 | + | 51.02 | 16.67 | 46.96 | + | + | - | 41.67 |
| Pambali | 9.68 | + | 31.18 | 10.5 | 6.90 | 50.39 | 20.00 | 10.64 | + | - | + | 39.13 |
| Pulicat | 17.39 | 73.68 | 16.42 | 10.77 | + | 41.61 | 8.52 | + | + | + | + | 50.00 |
| Broken Bridge | + | 64.29 | 9.38 | 13.04 | + | 86.96 | 5.71 | + | - | + | - | 63.16 |
| Neelangarai | 20.69 | + | 22.6 | 26.8 | + | + | + | + | - | 14.29 | - | 47.83 |
| Vayalur | + | + | 6.40 | 13.87 | + | + | + | + | + | 4.35 | - | 52.00 |
| Pondicherry | + | - | 10.96 | 0.00 | - | + | + | 0.00 | - | + | - | 31.58 |
| Singarathope | + | + | 9.43 | 3.19 | + | + | + | 6.90 | + | + | - | 40.00 |
| Thirumullaivasal | + | + | 4.14 | 3.39 | + | - | + | + | - | 1.75 | - | 23.53 |
| Karaikal | + | - | 12.97 | 11.76 | 2.82 | - | 20.69 | - | + | 2.27 | - | 33.33 |
| VKP Beach | - | - | + | 25.19 | 14.71 | - | 0.00 | - | + | + | - | 36.36 |
| Kodiyakarai | - | - | 65.45 | 65.05 | 0.00 | - | + | 30.99 | + | 5.15 | + | 36.67 |
| Gopalpattinam | - | + | + | + | - | - | + | + | 0.00 | - | - | 14.29 |
| Athiyuthur | + | + | 1.49 | - | + | - | + | - | + | - | - | 25.00 |
| Dhanushkodi | 14.29 | + | 26.02 | + | + | 42.7 | 15.87 | + | + | + | - | 42.86 |
| Alavangulam | 33.33 | 25.00 | 29.66 | 9.52 | + | 25.35 | 62.86 | + | + | + | - | 41.67 |
| Muthu Nagar | + | + | 0.00 | + | - | + | + | + | + | - | - | 11.54 |
| Manapadu | + | - | 12.72 | 8.11 | + | 31.48 | - | + | + | + | - | 30.43 |
| Avudaiyalpuram | + | - | 0.00 | 0.00 | + | + | + | + | - | + | - | 6.25 |
